# Supplementary material for: Deep phenotypic profiling of neuroactive drugs in larval zebrafish
Source: Nat Commun. 2024 Nov 17;15:9955. doi: 10.1038/s41467-024-54375-y (PMC11570628; doi:10.1038/s41467-024-54375-y)
Supplement: Supplementary file 1 — Supplementary Information [file 41467_2024_54375_MOESM1_ESM.pdf]

Supplementary Material for
Deep phenotypic profiling of neuroactive drugs in larval zebrafish

Leo Gendelev[1], Jack Taylor[1,2], Douglas Myers-Turnbull[1], Steven Chen[3], Matthew N. McCarroll[1,3], Michelle R. Arkin[3], David Kokel[1 *], Michael J. Keiser[1,3,4,5 *]

1. Institute for Neurodegenerative Diseases, University of California, San Francisco, San Francisco, CA, USA
2. UCSF Weill Institute for Neurosciences Memory and Aging Center, University of California, San Francisco, CA, USA
3. Department of Pharmaceutical Chemistry, University of California, San Francisco, San Francisco, CA, USA
4. Department of Bioengineering and Therapeutic Sciences, University of California, San Francisco, CA, USA
5. Bakar Computational Health Sciences Institute, University of California, San Francisco, San Francisco, CA, USA

* Correspondence to: dave.kokel@gmail.com, keiser@keiserlab.org

Supplementary Figure 1

a. Toxic - Butaclamol

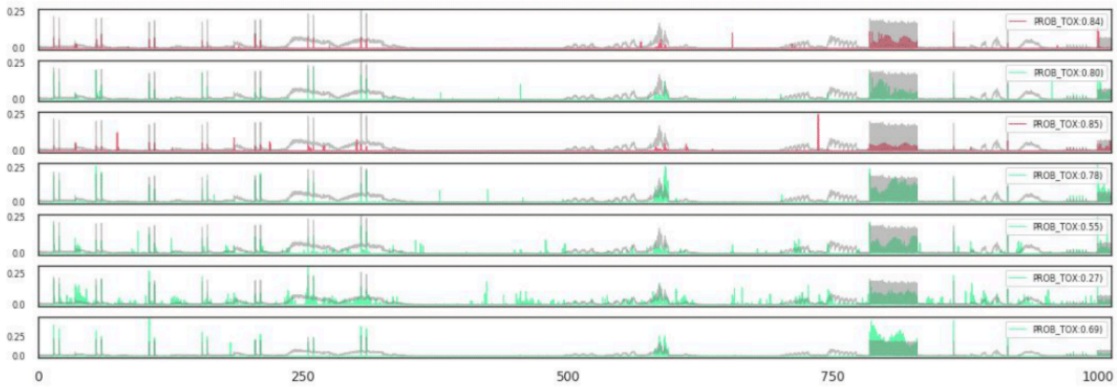

b. Inactive - 2-PMDQ

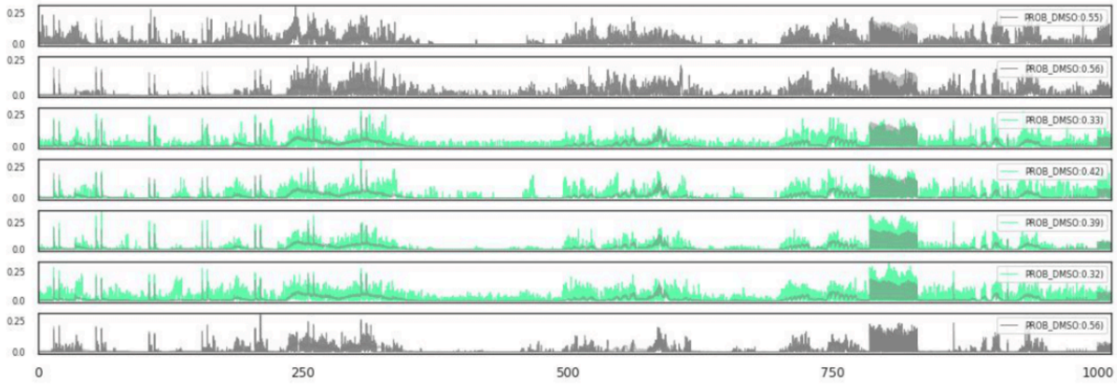

c. Active - Yohimbine

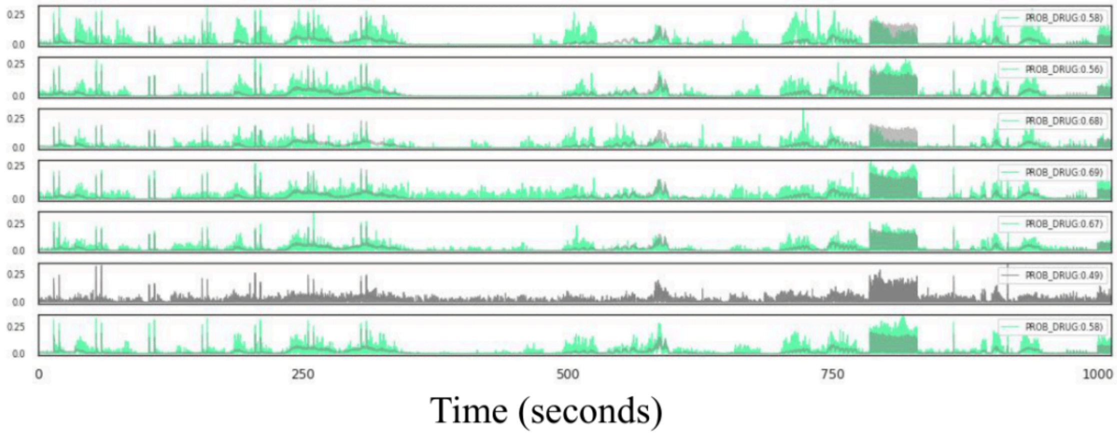

Random forest classification examples. Examples of using the random forest classifier to bin NT-650 drugs into three categories: active, inactive, and toxic. (a) Toxic example (Butaclamol). 2 of the 7 traces have a toxic probability > 0.8 (red lines), so we classify this drug as toxic. (b) Inactive example (2-MPDQ). 3 of the 7 traces have an inactive or "DMSO" probability > 0.5 (grey lines), so we classify this drug as inactive. (c) Active examples (Yohimbine). 6 of the 7 traces have a drug probability > 0.5 (green lines), so we classify this drug as active. All motion index time series y-axis are plotted on a minimum and maximum scale of 0 to 1 based on the minimum and maximum of the dataset which are 0 and 6750, respectively. Source data are provided in the Source Data File.

Supplementary Figure 2

a. Random Features (MI Values)

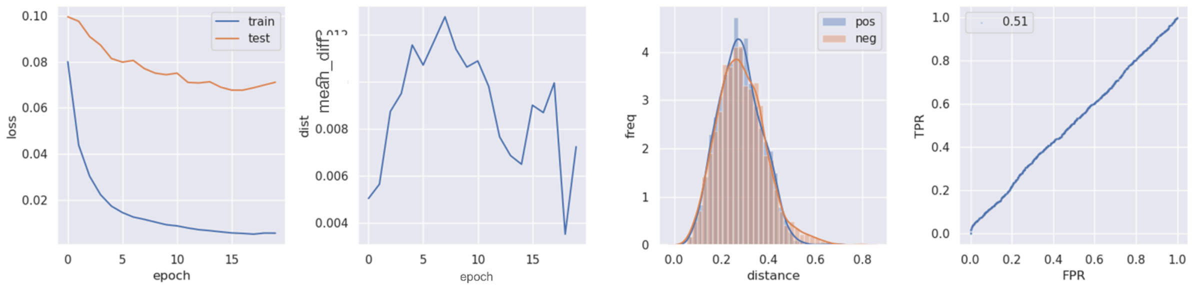

b. Randomized Labels

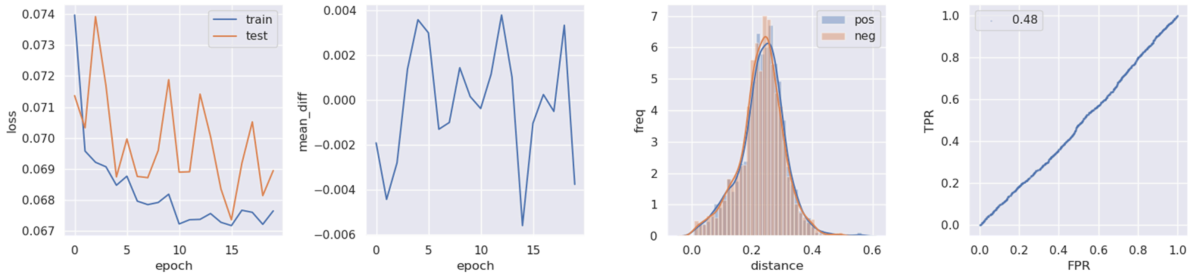

c. Predicting well dist (neighbor cutoff 5.2)

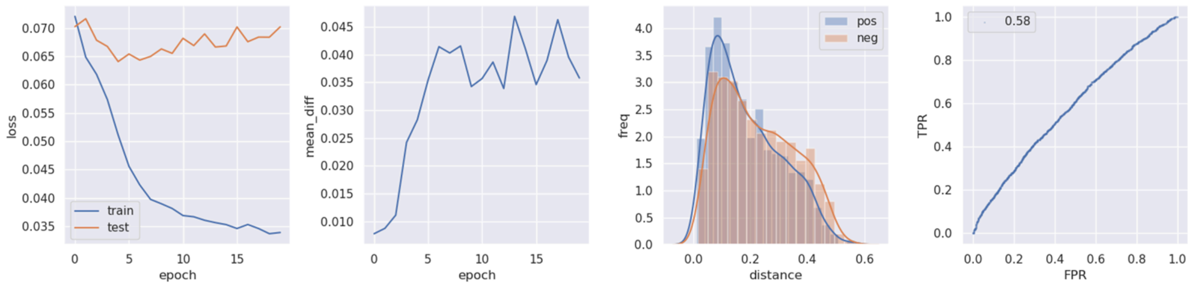

d. Predicting well dist (neighbor cutoff 2.0)

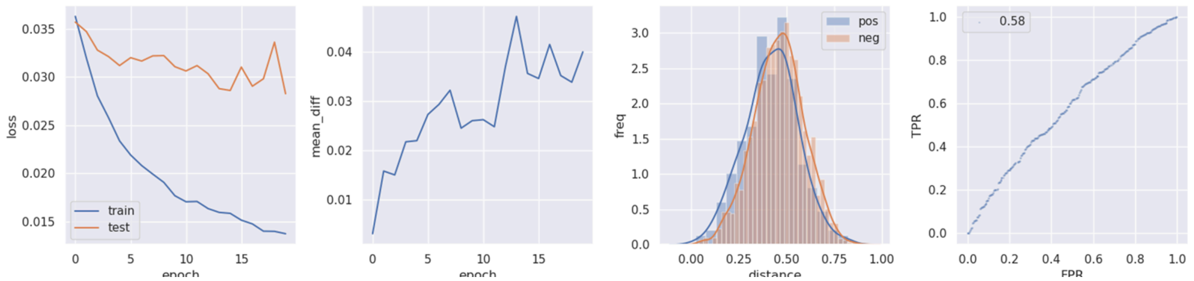

Model soundness checks. We perform a set of soundness checks for the fully randomized screen. (a) We train the Twin-NN model with completely randomized input features (motion index time series) and (b) with randomized output labels. In both cases, the result is a model that cannot distinguish between positive and negative pairs. (c) We train Twin-NN models where positive and negative pairs are defined by plate distance within a neighbor-cutoff of 2.0 and (d) with a neighbor-cutoff of 5.0. If a pair is within the neighbor-cutoff distance, it is a positive pair and negative otherwise. In both cases, these models cannot distinguish positive from negative pairs. Source data are provided in the Source Data File.

Supplementary Figure 3

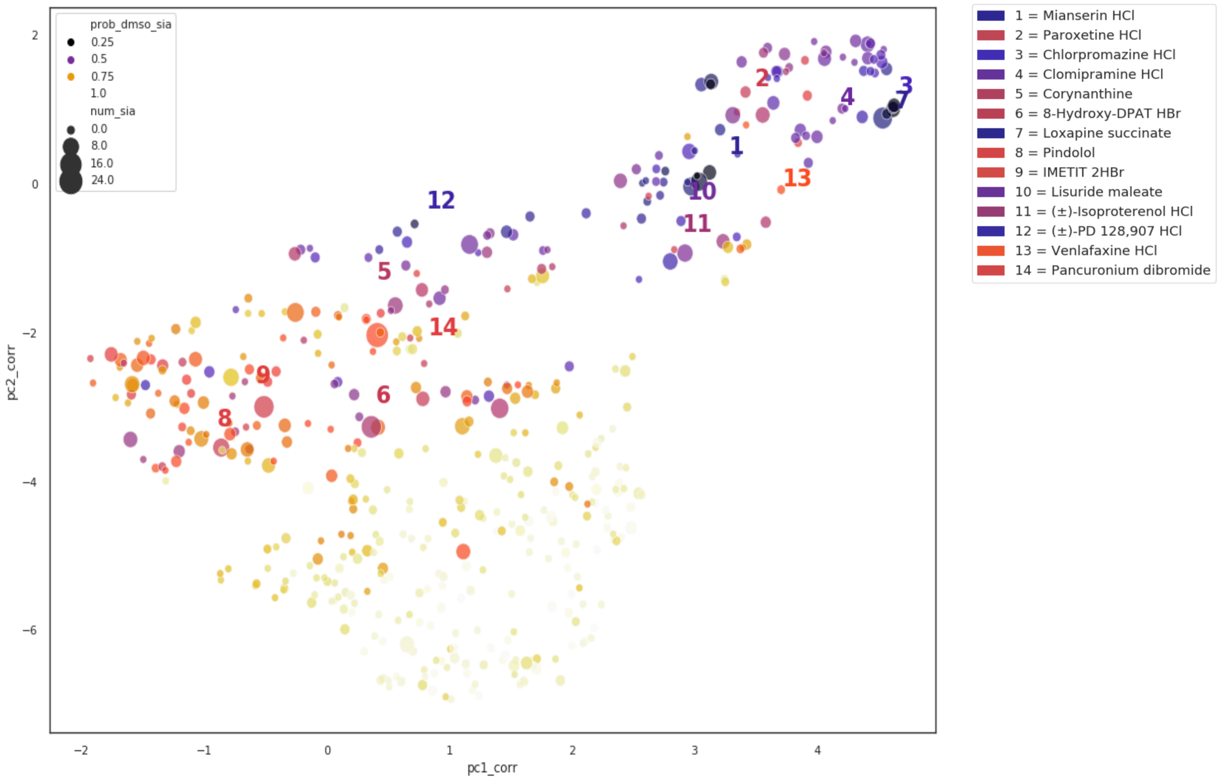

Correlation distance behaviorome UMAP. Same as Figure 4 (Results), but using correlation distance. Source data are provided in the Source Data File.

Supplementary Figure 4

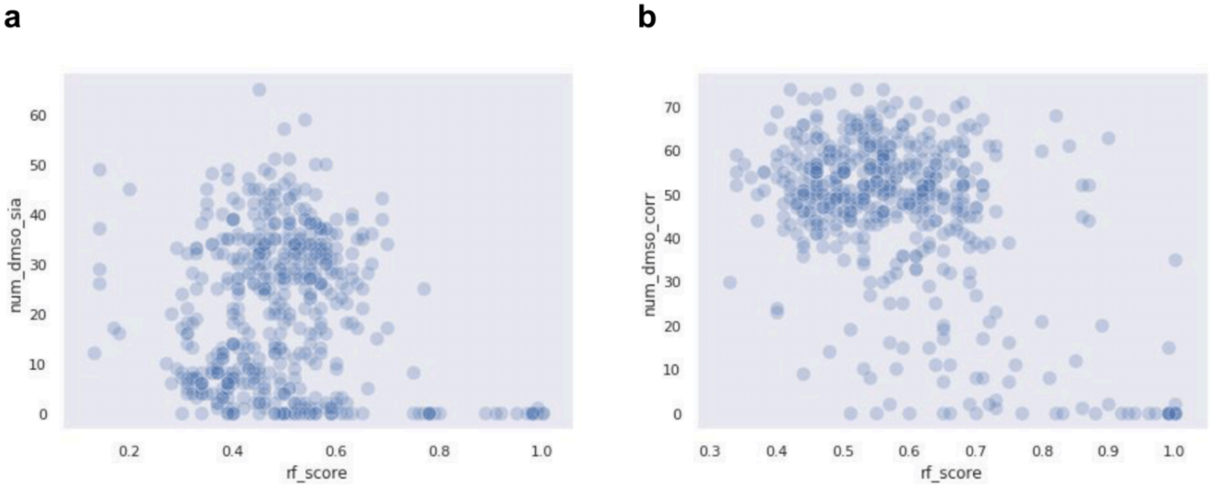

Correlation distance vs Twin-NN "phenosearch" top-ranked control-well counts. We plot the number of control (DMSO) wells (y-axis) in the pheno-search results for the NT-650 compounds compared with the strength of the phenotype (rf_score, x-axis) (a) using Twin-NN distance for the phenosearch (b) using correlation distance. Strikingly, there is a highly populated area of points with Twin-NN, average-strength rf_score (0.2-0.5) with few control wells, whereas very few points of this type with correlation distance. This suggests Twin-NN distance is more effective at finding drug-like DIVERSet compounds in pheno-searches than correlation distance, especially for compounds with weaker or average-strength phenotypes. Source data are provided in the Source Data File.

Supplementary Figure 5

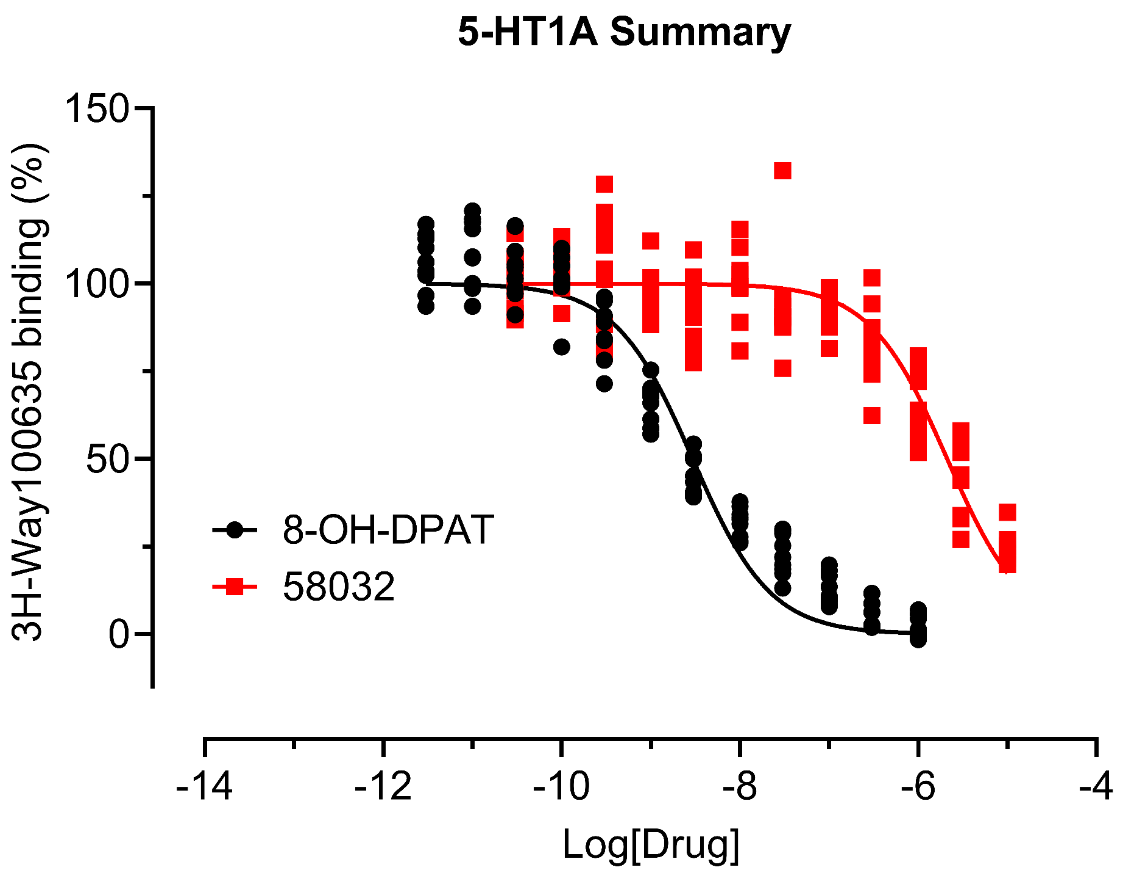

5-HT1A Secondary Binding Curves. Results (mean ± SEM) from a minimum of 3 independent assays (each in triplicate) were normalized, pooled, and analyzed using the built-in competition binding function in the GraphPad Prism V10. Source data are provided in the Source Data File.

Supplementary Figure 6

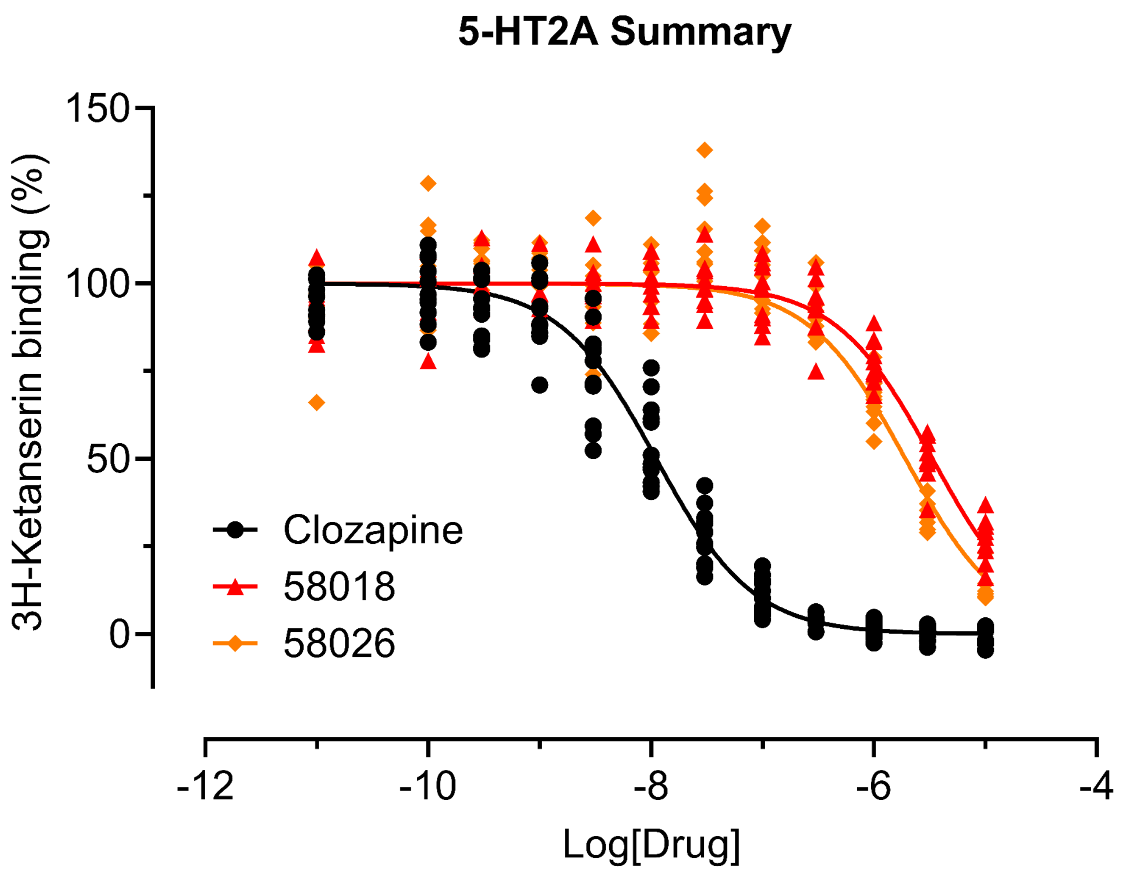

5-HT2A Secondary Binding Curves. Results (mean ± SEM) from a minimum of 3 independent assays (each in triplicate) were normalized, pooled, and analyzed using the built-in competition binding function in the GraphPad Prism V10. Source data are provided in the Source Data File.

Supplementary Figure 7

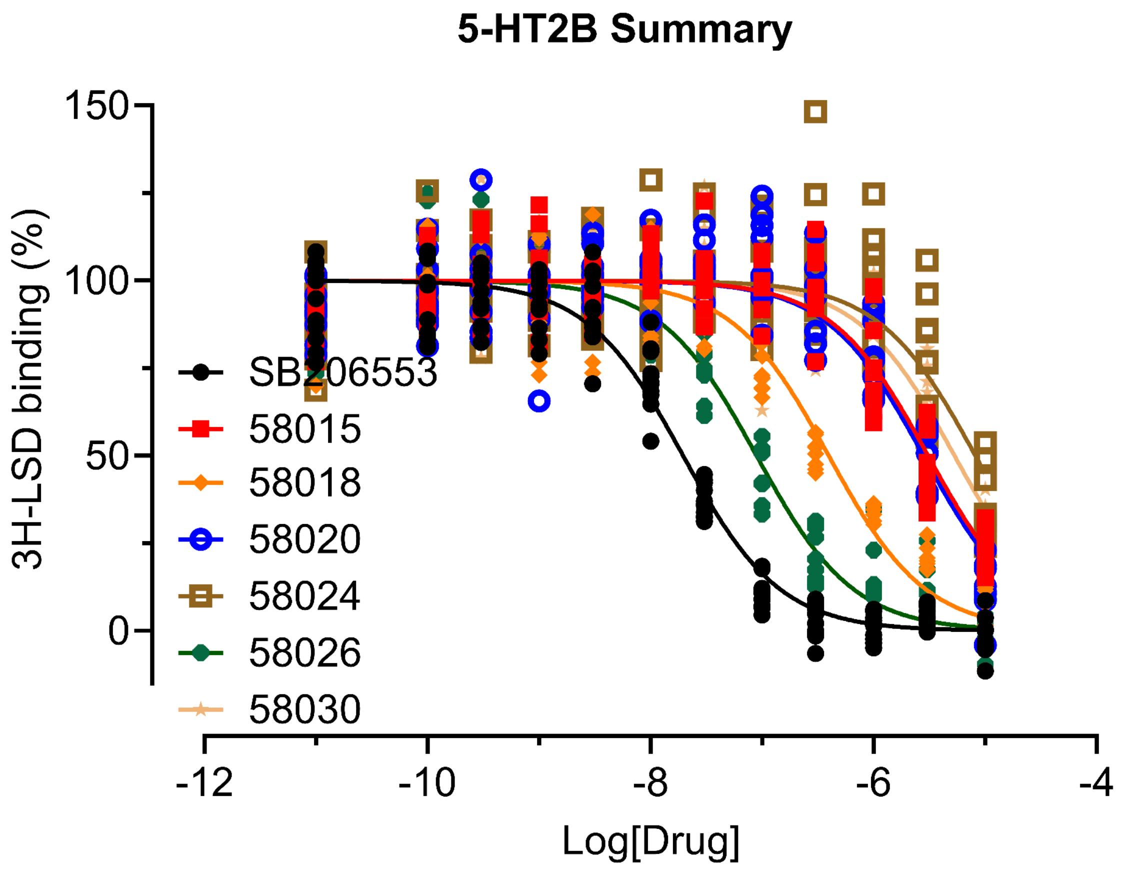

5-HT2B Secondary Binding Curves. Results (mean ± SEM) from a minimum of 3 independent assays (each in triplicate) were normalized, pooled, and analyzed using the built-in competition binding function in the GraphPad Prism V10. Source data are provided in the Source Data File.

Supplementary Figure 8

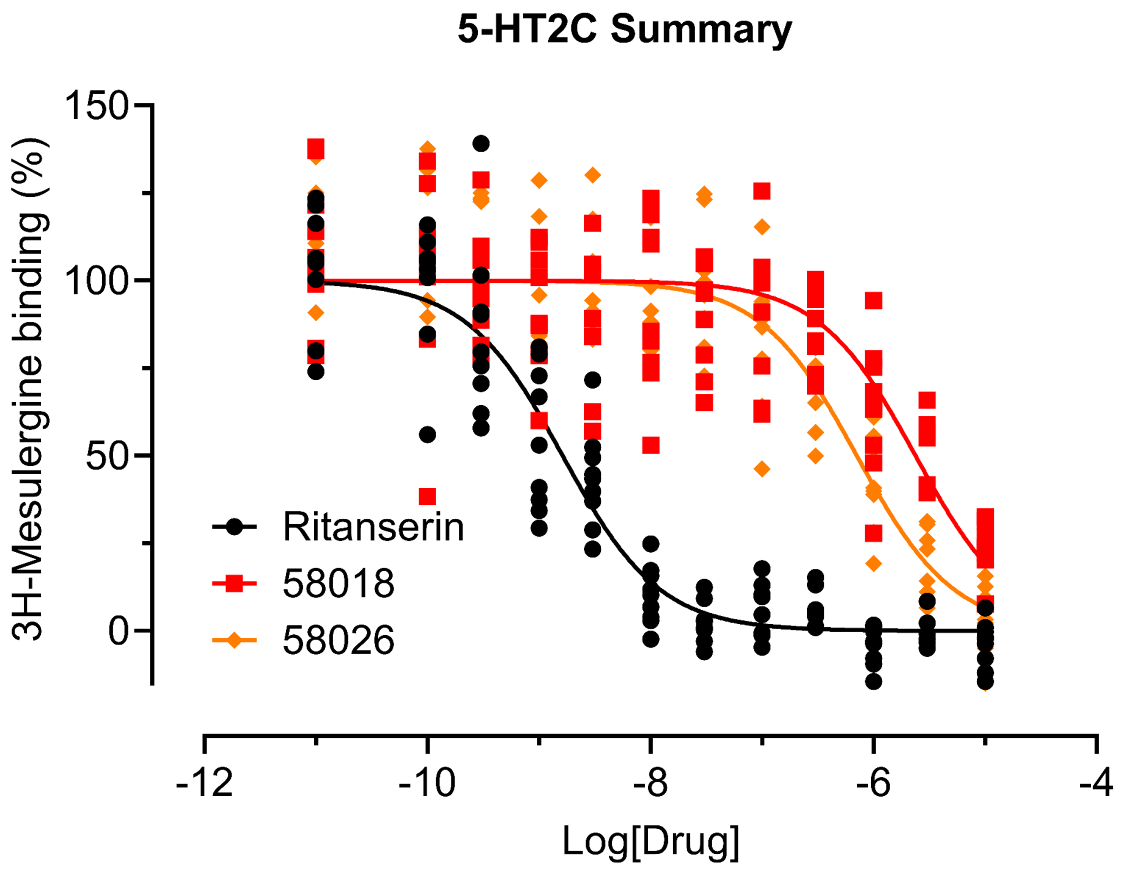

5-HT2C Secondary Binding Curves. Results (mean ± SEM) from a minimum of 3 independent assays (each in triplicate) were normalized, pooled, and analyzed using the built-in competition binding function in the GraphPad Prism V10. Source data are provided in the Source Data File.

Supplementary Figure 9

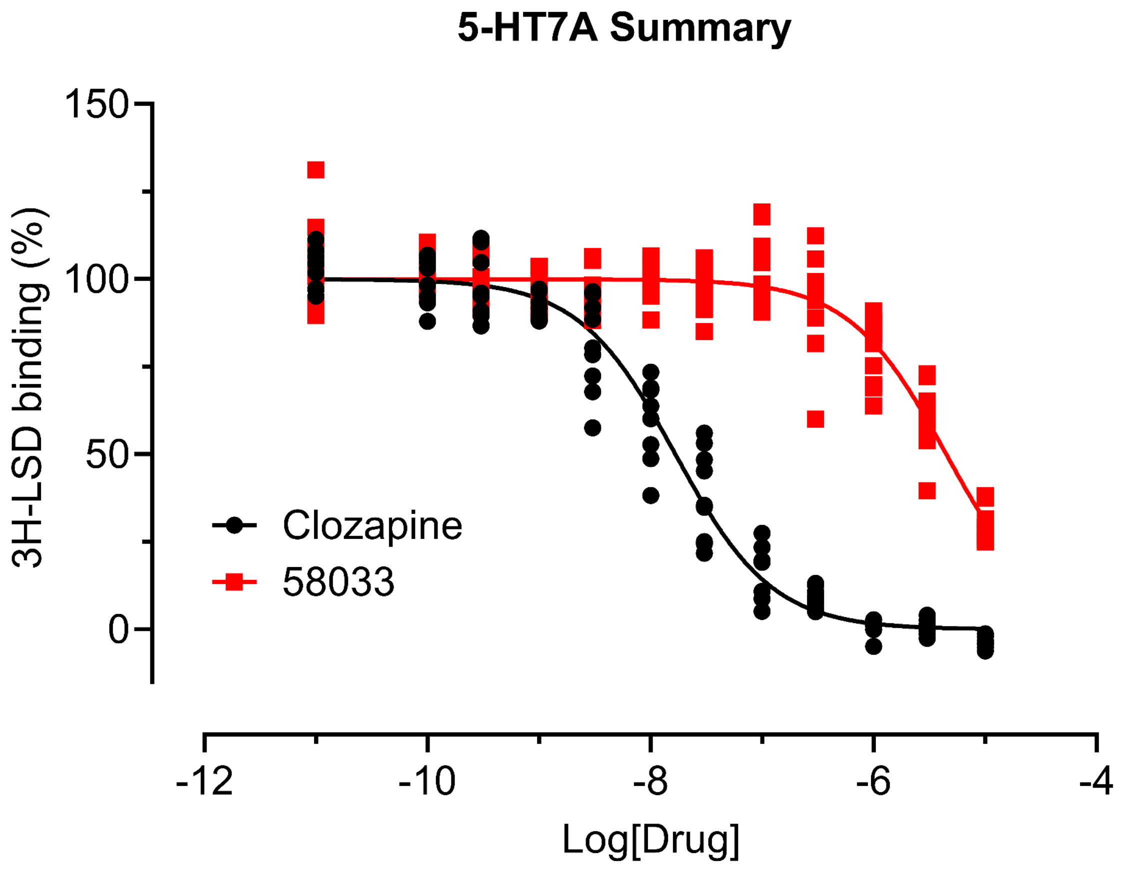

5-HT7A Secondary Binding Curves. Results (mean ± SEM) from a minimum of 3 independent assays (each in triplicate) were normalized, pooled, and analyzed using the built-in competition binding function in the GraphPad Prism V10. Source data are provided in the Source Data File.

Supplementary Figure 10

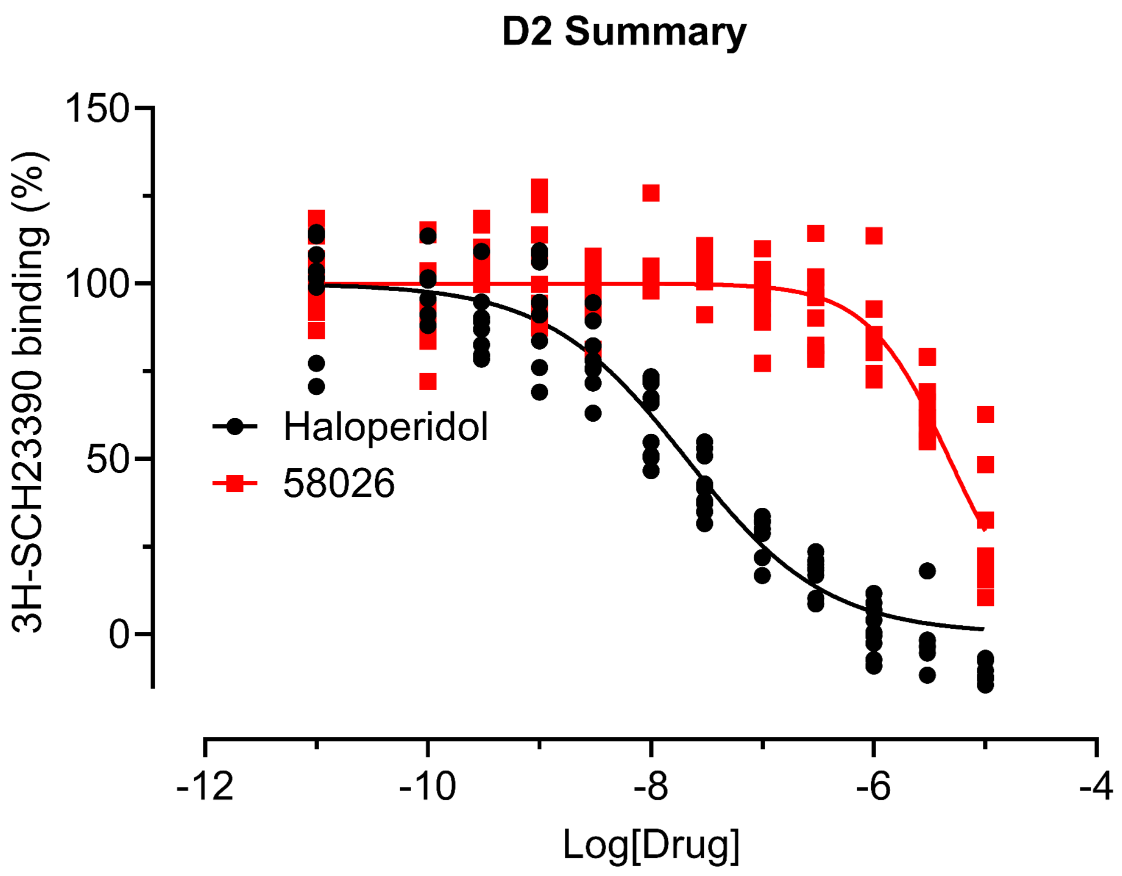

Dopamine D2 Receptor Secondary Binding Curves. Results (mean ± SEM) from a minimum of 3 independent assays (each in triplicate) were normalized, pooled, and analyzed using the built-in competition binding function in the GraphPad Prism V10. Source data are provided in the Source Data File.

Supplementary Figure 11

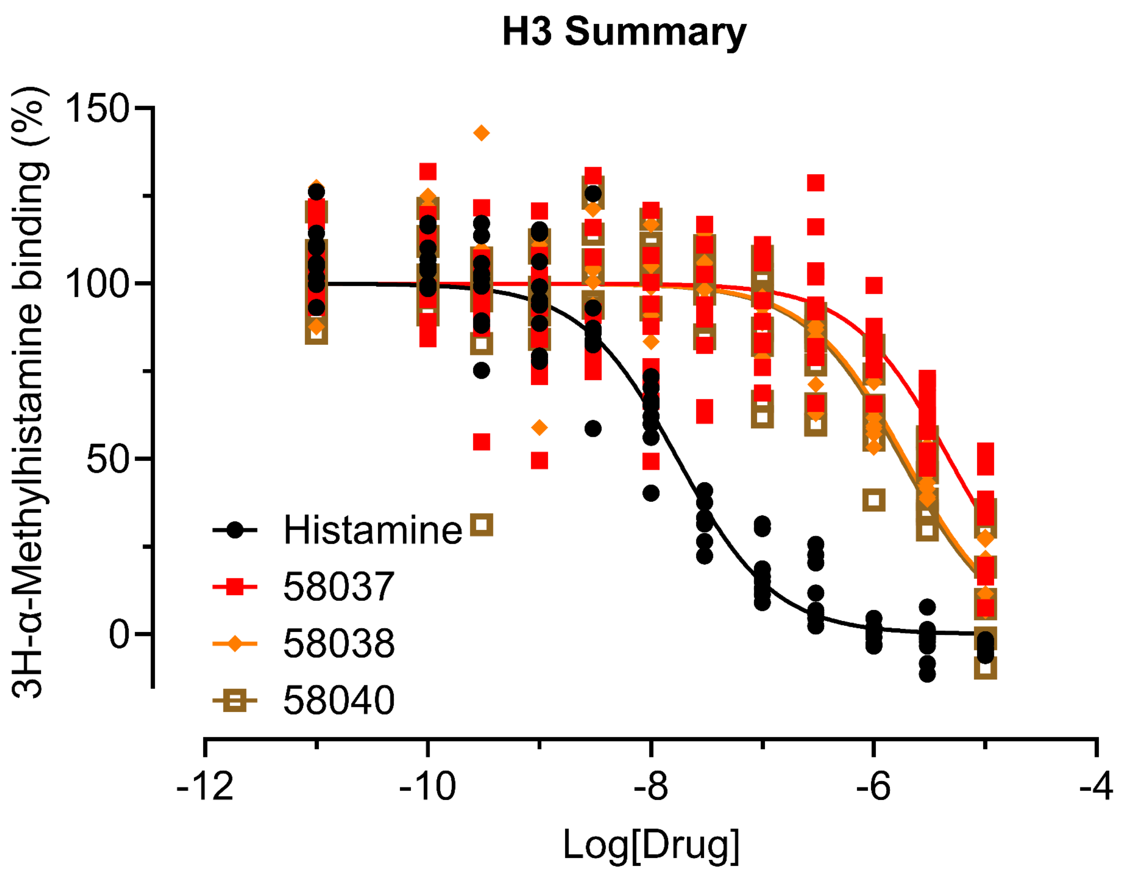

Histamine H3 Receptor Secondary Binding Curves. Results (mean ± SEM) from a minimum of 3 independent assays (each in triplicate) were normalized, pooled, and analyzed using the built-in competition binding function in the GraphPad Prism V10. Source data are provided in the Source Data File.

Supplementary Figure 12

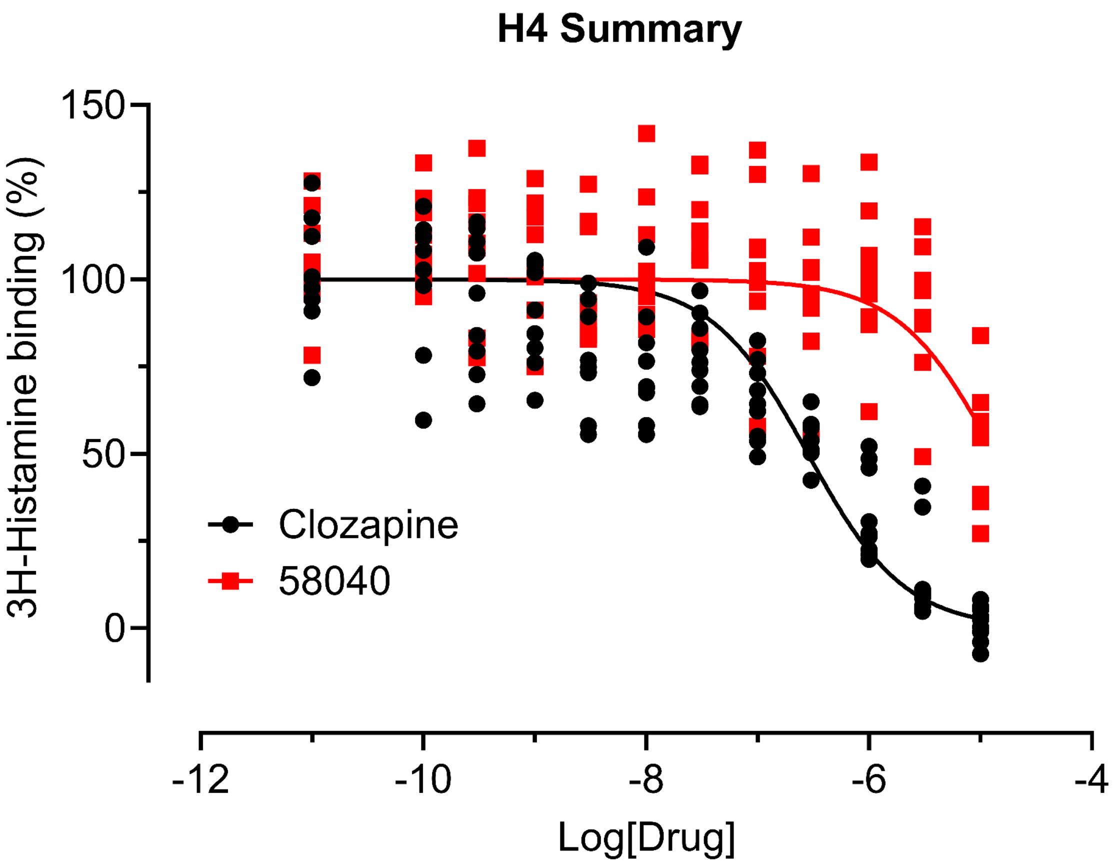

Histamine H4 Receptor Secondary Binding Curves. Results (mean ± SEM) from a minimum of 3 independent assays (each in triplicate) were normalized, pooled, and analyzed using the built-in competition binding function in the GraphPad Prism V10. Source data are provided in the Source Data File.

Supplementary Figure 13

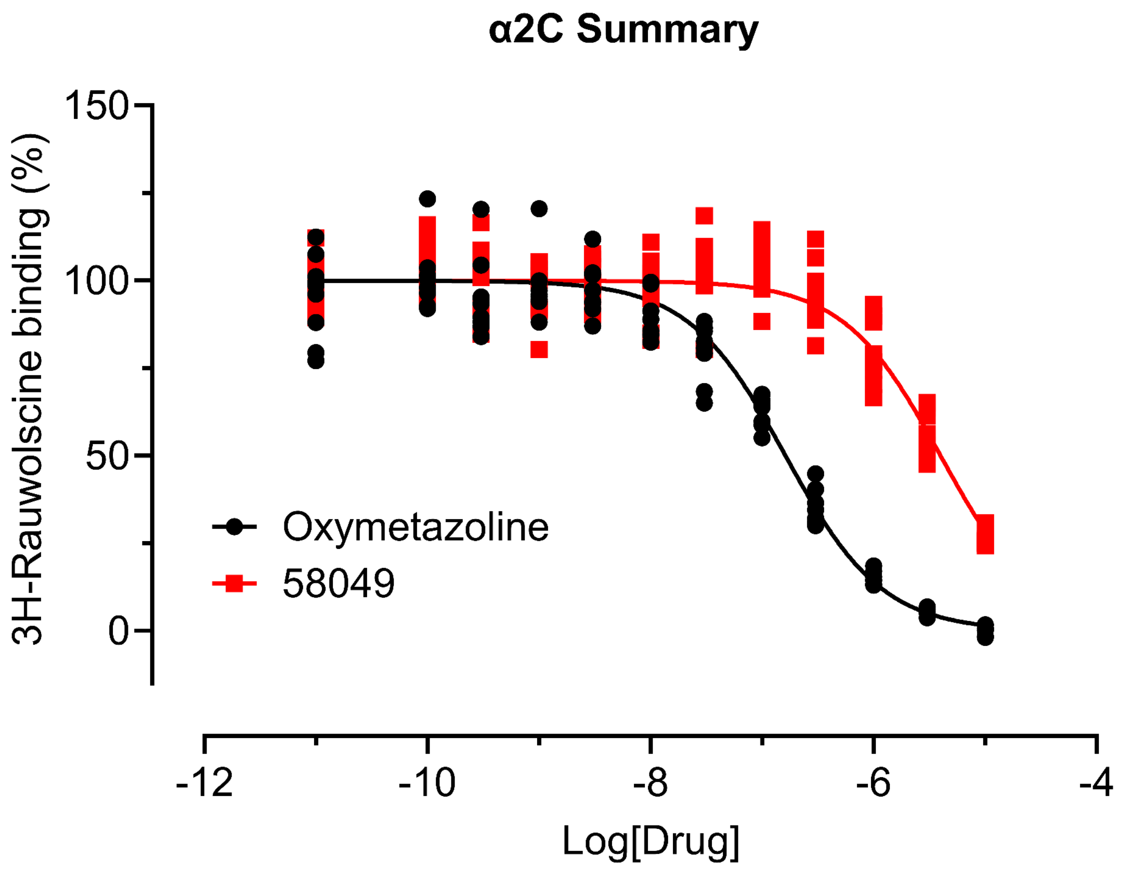

Alpha 2C Adrenergic Receptor Secondary Binding Curves. Results (mean ± SEM) from a minimum of 3 independent assays (each in triplicate) were normalized, pooled, and analyzed using the built-in competition binding function in the GraphPad Prism V10. Source data are provided in the Source Data File.

Supplementary Figure 14

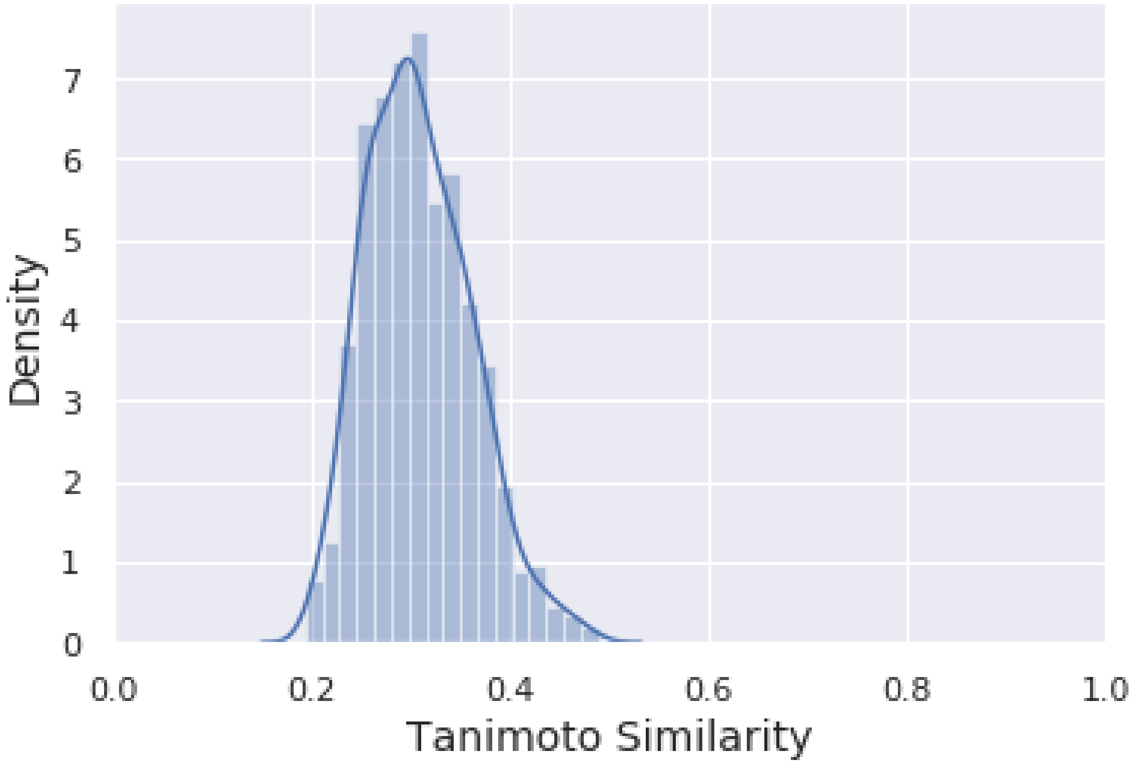

NT650 vs DIVERSet chemical similarities. For each drug in the NT-650 set, we compute its Tanimoto similarity to the top 50 DIVERSet compounds from the Twin-NN phenoblast. We plot the distribution of mean similarity scores across all NT-650 drugs. Source data are provided in the Source Data File.

Supplementary Figure 15

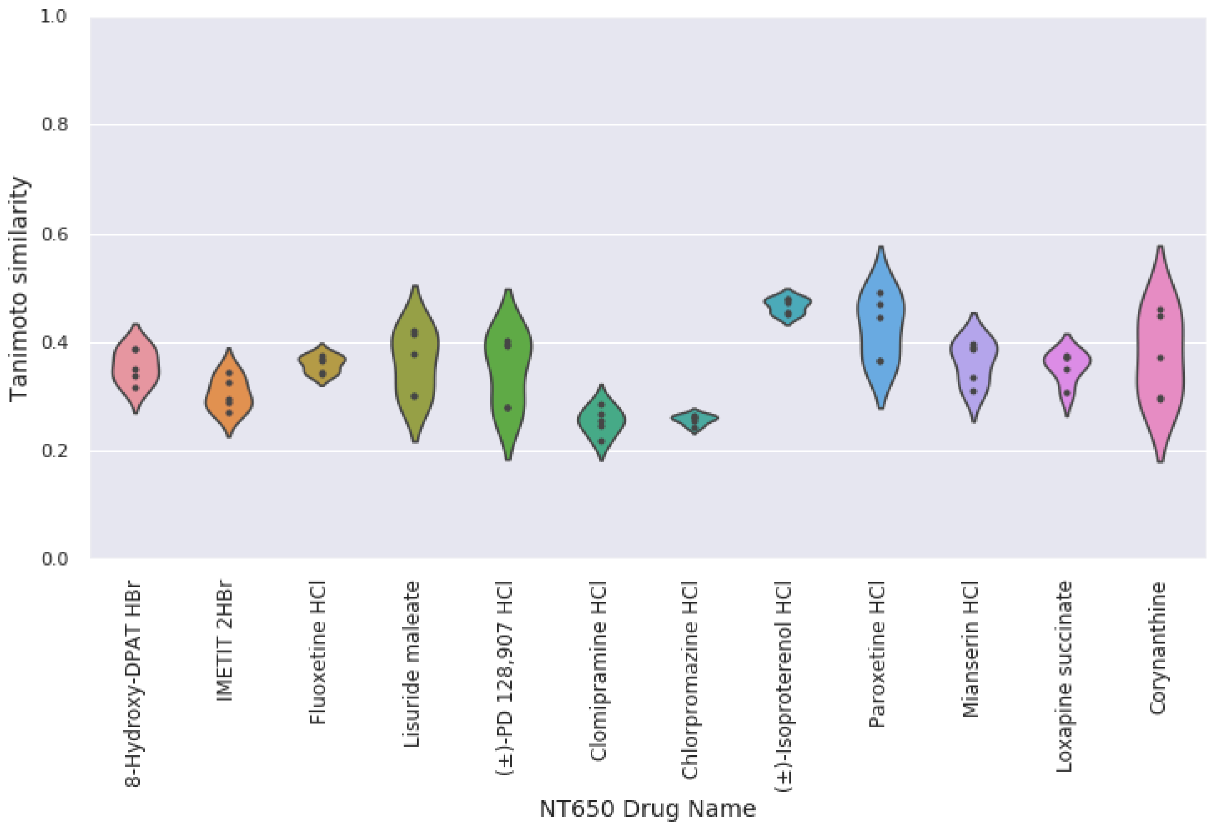

Chemical similarities of experimentally tested DIVERSet compounds to NT650 drugs. For all experimentally tested DIVERSet compounds linked by phenosearch to query NT-650 compounds, we show violin plots based on the Tanimoto similarity of the top 5 phenomatched DIVERSet compounds compared against the associated NT-650 drug queries. We computed Tanimoto similarities based on ECFP4 fingerprints and generated the violin plots using the python-seaborn package. Source data are provided in the Source Data File.

Supplementary Figure 16

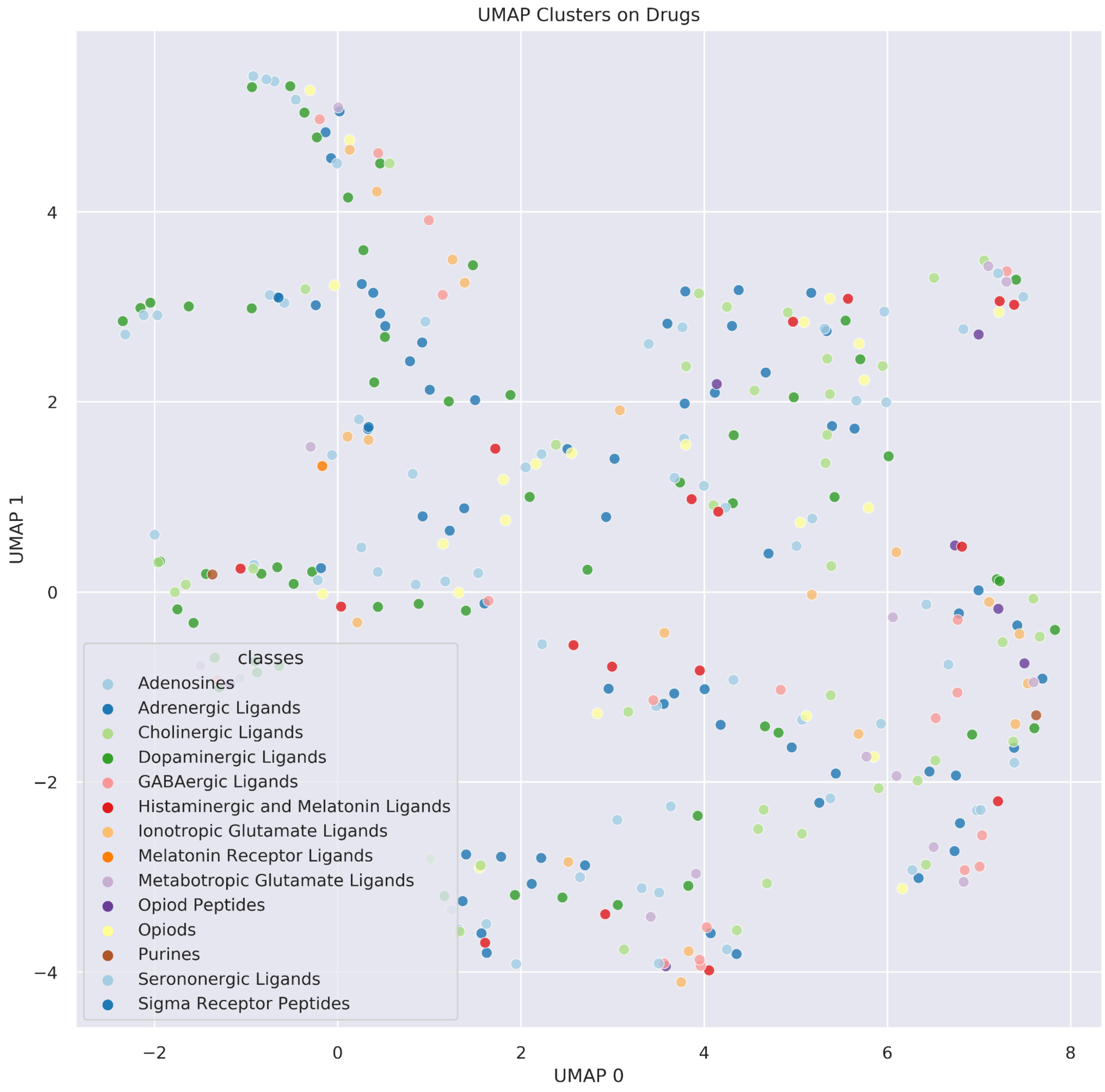

Behaviorome with supplier-provided "Classes" for drugs predicted to have strong phenotypes (n= 325 drugs; 50% of NT650). We used supplier-provided plate classes (14 total) for the NT-650 drugs, removed compounds without strong phenotypes according to the random forest drug classifier, and plotted a UMAP behaviorome for the 325 remaining compounds. We colored compounds by the 14 plate class labels. Source data are provided in the Source Data File.

Supplementary Figure 17

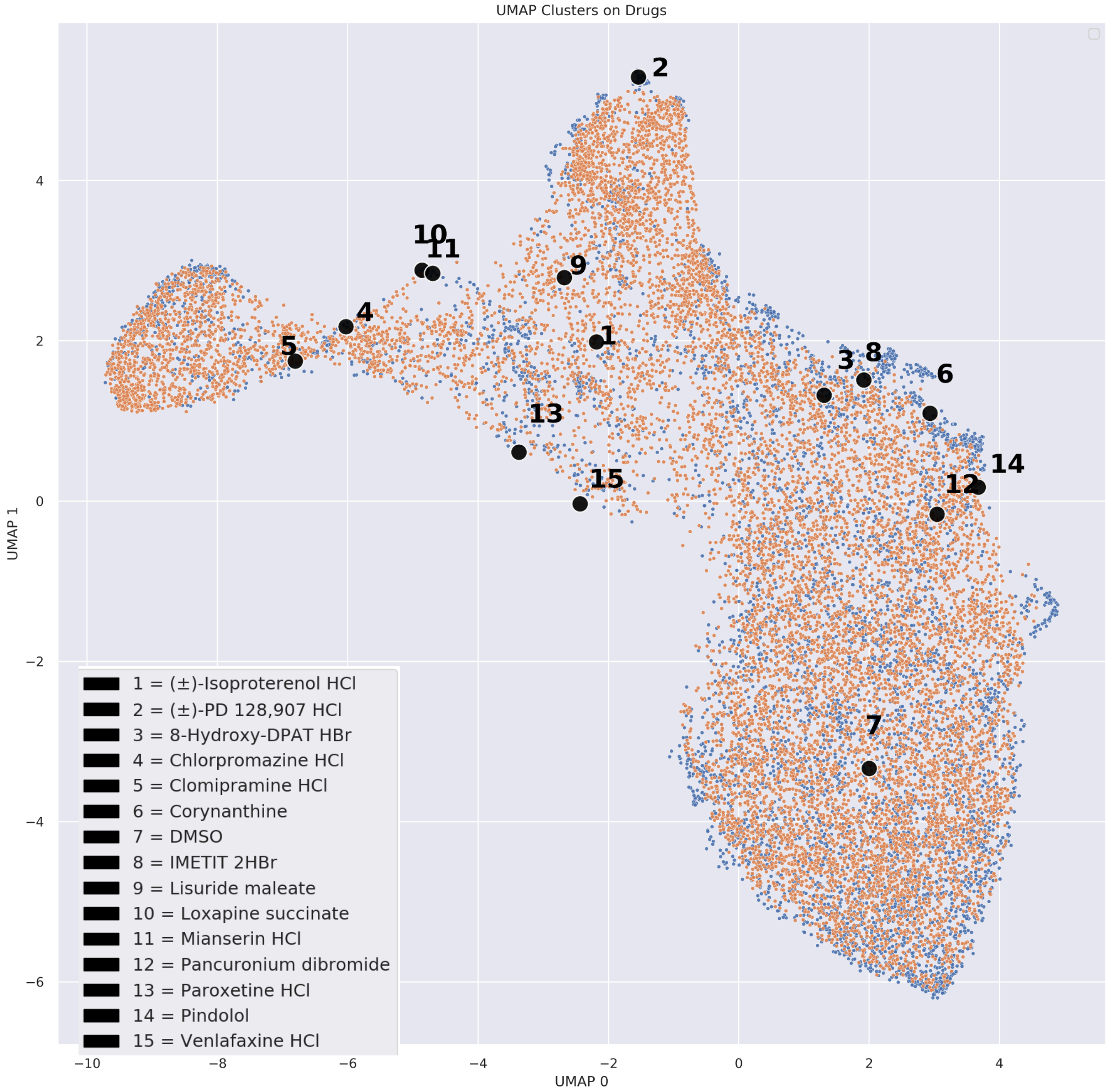

Complete Behaviorome (NT650 and DIVERSet) overlay. A combined behaviorome based on all individual replicates of NT-650 drugs and all DIVERSet compounds taken together. Compounds are colored by dataset. Markers correspond to the same drugs as in the NT-650 behaviorome (Figure 4) for reference. Source data are provided in the Source Data File.

Supplementary Table 1. Internal ID to Hit2Lead compound ID map

Internal_ID	Hit2Lead_ID
c29237	9210449
c26692	9239432
c29296	9212284
c25328	9207145
c26732	9240531
c33446	9302621
c33448	9306584
c29676	9216089
c33858	9336481
c29440	6423322
c32656	9275801
c26429	9232516
c33965	9343970
c33151	9284190
c31211	9247328
c25518	9211046
c27884	9265565
c25477	9209700
c24594	4033665
c31386	9251970
c34037	9201123
c33553	9308643
c33255	9287933
c31508	9254844
c31447	9253440
c28263	9277938
c33507	9309842
c26398	9232267
c26521	9232718

c33914	9340973
c30977	9243029
c28263	9277938
c27284	9253599
c33507	9309842
c26398	9232267
c33623	9317255
c25826	9218579
c30164	9226137
c25822	9217785
c29886	9220595
c32314	9268194
c24681	4035747
c33331	9289775
c30141	9226742
c26283	9230064
c24969	9193557
c32339	9270758
c31000	9244148
c25173	9203272
c25237	9203845
c30840	9239850
c30882	9239370
c29623	9195413
c28359	9278979
c30872	9239357
c26167	9225879
c32103	9265563
c33329	9293951
c28262	9277579
c27280	9253048

Supplementary Table 2. Primary assays (%-inhibition values)

Drug Query	Compound ID	PDSP ID	SERT	NET	5-HT2A	5-HT2C	
Fluoxetine	c29237	57994	-28.79	-8.73	10.26	-1.78	
	c26692	57995	-30.52	21.02	3.75	-5.15	
	c29296	57996	-30.52	21.5	15.27	4.32	
	c25328	57997	-26.76	35.82	22.15	5.24	
	c26732	57998	-15.64	-2.57	-1.92	-1.18	
Paroxetine	c33446	57999	-25.87	11.05	12.5	5.69	
	c33448	58000	-20.59	1.69	3.75	-1.08	
	c29676	58001	-24.45	-6.15	24.79	10.4	
	c33858	58002	-28.43	-16.62	8.26	20.12	
	c29440	58003	-22.69	-6.55	8.63	1.72	
			D2	D3	5-HT2A	5-HT2C	
Lisuride	c32656	58004	15.77	-3.78	45.23	-6.1	
	c26429	58005	17.55	5.08	-6.43	2.32	
	c33965	58006	15.9	-1.07	1.75	4.64	
	c33151	58007	26.43	3.85	-4.56	-0.6	
	c31211	58008	23.13	-0.78	-14.94	-8.81	
(±) PD 128,907 HCl	c25518	58009	8.46	-2.37	-9.32	-1.02	
	c27884	58010	19.52	8	1.59	-9.82	
	c25477	58011	18.82	12.67	-3.42	-9.89	
	c24594	58012	11.29	-8.25	-10	-6.8	
	c31386	58013	17.17	0.8	-3.94	-0.61	
			5-HT2A	5-HT2B	5-HT2C	D1	D2
Chlorpromazine	c34037	58014	8.75	33.64	31.01	28.4	18.35
	c33553	58015	12.77	67.79	41.05	17.82	7.66
	c33255	58016	25.9	48.66	26.7	12.5	6.64
	c31508	58017	3.85	3.23	-19.39	0.09	7.31
	c31447	58018	61.94	86.4	59.09	25.91	39.24
Mianserine	c28263	58019	6.74	-6.2	7.12	-17.1	6.22
	c33507	58020	28.46	75.25	3.16	-3.69	21.02

	c26398	58021	6.07	13.94	5.57	-19.09	25.88
	c26521	58022	-7.87	-2.8	5.87	-3.8	2.37
	c33914	58023	-2.97	24.18	2.75	-6.73	15.17
Loxapine	c30977	58024	7.2	50.77	6.67	-0.42	28.22
	c28263	58019	6.74	-6.2	7.12	-17.1	6.22
	c27284	58025	-8.12	-10.13	-4.12	-3.74	14.83
	c33507	58020	28.46	75.25	3.16	-3.69	21.02
	c26398	58021	6.07	13.94	5.57	-19.09	25.88
Clomipramine	c33623	58026	78.39	92.17	82.11	40.26	67.55
	c25826	58027	-4.81	29.57	1.24	-7.56	24.41
	c30164	58028	6.7	27.58	-4.62	-5.63	16.78
	c25822	58029	0.92	0.58	-8.87	-4.85	14.71
	c29886	58030	7.41	51.77	13.86	9.62	20.43

			5-HT1A	5-HT7A	D2
8-Hydroxy-DPAT	c32314	58031	4.54	40.03	16.15
	c24681	58032	63.5	40.97	16.91
	c33331	58033	11.69	51.18	19.7
	c30141	58034	-2.36	39.83	1.06
	c26283	58035	-4.38	28.38	38.05
			H3	H4	
IMETIT 2HBr	c24969	58036	8.14	12.44	
	c32339	58037	50.2	38.18	
	c31000	58038	68.59	63.29	
	c25173	58039	33.04	6.94	
	c25237	58040	86.54	9.11	
			Beta1	Beta2	Beta3
Isoproteronol	c30840	58041	10.83	#N/A	#N/A
	c30882	58042	12.96	#N/A	#N/A
	c29623	58043	21.4	#N/A	#N/A
	c28359	58044	17.81	#N/A	#N/A
	c30872	58045	-0.3	#N/A	#N/A
			Alpha1A	Alpha2C	

Corynanthine	c26167	58046	#N/A	17.78
	c32103	58047	#N/A	17.16
	c33329	58048	#N/A	41.07
	c28262	58049	#N/A	63.58
	c27280	58050	#N/A	38.2

Cell shading progresses from largest negative value (pink) to largest positive value (orange).

Supplementary Table 3. Known-drug pairs related by phenotypic distance but with dissimilar structures

namex	namey	twin-nn dist	tanimoto dist	correlation dist	Enrichment over 2D	ChEMBL_ Similarity
7-Hydroxy-DPAT HBr	Ropinirole HCl	0.254	0.543	0.269	1.288	0.636
(±)-PD 128,907 HCl	Ropinirole HCl	0.233	0.567	0.275	1.335	0.556
(+)-PD 128907	Ropinirole HCl	0.224	0.567	0.277	1.343	0.556
trans-7-Hydroxy-PIPAT maleate	LY-163,502 2HCl	0.136	0.505	0.211	1.369	0.500
1-[1-(2-Benzo[b]thienyl)cyclohexyl)]piperidine maleate	PRE-084·HCl	0.268	0.574	0.234	1.306	0.500
Ropinirole HCl	Lisuride maleate	0.132	0.549	0.323	1.417	0.444
7-Hydroxy-DPAT HBr	Pergolide mesylate	0.257	0.576	0.168	1.319	0.438
Ropinirole HCl	trans-7-Hydroxy-PIPAT maleate	0.092	0.544	0.280	1.453	0.400
LY-163,502 2HCl	(±)-SKF-82958 HBr	0.228	0.534	0.229	1.306	0.400
LY-163,502 2HCl	R(+)-6-BROMO-APB HBr	0.192	0.529	0.326	1.337	0.400
Mianserin HCl	Oxotremorine sesuifumarate	0.285	0.833	0.164	1.547	0.378
Ropinirole HCl	LY-163,502 2HCl	0.103	0.578	0.249	1.475	0.375
trans-7-Hydroxy-PIPAT maleate	Lisuride maleate	0.146	0.514	0.175	1.367	0.375
Yohimbine HCl	Oxymetazoline HCl	0.147	0.548	0.150	1.401	0.375
7-Hydroxy-DPAT HBr	Lisuride maleate	0.254	0.579	0.191	1.325	0.364
Clozapine	Chlorpromazine HCl	0.227	0.546	0.248	1.319	0.337
Clozapine	Chlorpromazine HCl	0.215	0.546	0.214	1.331	0.337
7-Hydroxy-DPAT HBr	Bromocriptine mesylate	0.187	0.594	0.323	1.408	0.333
7-Hydroxy-DPAT HBr	LY-163,502 2HCl	0.249	0.534	0.173	1.285	0.300
trans-7-Hydroxy-PIPAT maleate	R(-)-Propylnorapomorphine HCl	0.277	0.523	0.350	1.246	0.300
Mianserin HCl	Chlorpromazine HCl	0.199	0.538	0.136	1.339	0.288
Bromocriptine mesylate	Ropinirole HCl	0.155	0.538	0.340	1.382	0.286
Mianserin HCl	WB 4101 HCl	0.228	0.714	0.170	1.486	0.283
Chlorpromazine HCl	(+)-Butaclamol HCl	0.147	0.530	0.181	1.383	0.279
Chlorpromazine HCl	Pergolide mesylate	0.217	0.554	0.222	1.337	0.268

trans-7-Hydroxy-PIPAT maleate	Pergolide mesylate	0.290	0.542	0.190	1.252	0.267
Clothiapine	Thioridazine HCl	0.287	0.502	0.290	1.215	0.263
cis-(±)-N-methyl-N-[2-(3,4-dichlorophenyl)ETHYL]-2-(1-pyrrolidinyl)cyclohexamine	1-[1-(2-Benzo[b]thienyl)cyclohexyl)]piperidine maleate	0.192	0.610	0.163	1.419	0.250
cis-(±)-N-methyl-N-[2-(3,4-dichlorophenyl)ETHYL]-2-(1-pyrrolidinyl)cyclohexamine	PRE-084·HCl	0.242	0.687	0.206	1.445	0.250
6-Nitroquipazine maleate	Bopindolol malonate	0.140	0.603	0.261	1.463	0.250
PNU 96415E	trans-7-Hydroxy-PIPAT maleate	0.232	0.560	0.318	1.328	0.250
B-HT 920 2HCl	LY-163,502 2HCl	0.149	0.585	0.160	1.435	0.250
Ropinirole HCl	R(-)-Propylnorapomorphine HCl	0.256	0.538	0.380	1.282	0.250
trans-7-Hydroxy-PIPAT maleate	(±)-SKF-82958 HBr	0.257	0.514	0.228	1.257	0.250
trans-7-Hydroxy-PIPAT maleate	R(+)-6-BROMO-APB HBr	0.237	0.520	0.311	1.282	0.250
RS 17053 HCl	Naftopidil HCl	0.071	0.564	0.139	1.493	0.250
Clozapine	WB 4101 HCl	0.173	0.704	0.224	1.531	0.241
Clozapine	WB 4101 HCl	0.157	0.704	0.179	1.547	0.241
PNU 96415E	WB 4101 HCl	0.280	0.710	0.272	1.430	0.240
Cyproheptadine HCl	Mianserin HCl	0.280	0.559	0.390	1.279	0.239
Mianserin HCl	Clomipramine HCl	0.287	0.530	0.147	1.243	0.239
Bromocriptine mesylate	trans-7-Hydroxy-PIPAT maleate	0.167	0.534	0.336	1.366	0.231
Thioridazine HCl	Imipramine HCl	0.069	0.527	0.198	1.458	0.231
6-Nitroquipazine maleate	Nisoxetine HCl	0.175	0.702	0.180	1.527	0.222
PNU 96415E	B-HT 920 2HCl	0.155	0.658	0.266	1.503	0.222
SCH 23390 HCl	AMI-193	0.206	0.588	0.208	1.382	0.222
Mianserin HCl	Thioridazine HCl	0.293	0.505	0.205	1.212	0.217
WB 4101 HCl	RS 17053 HCl	0.258	0.603	0.261	1.346	0.217
Chlorpromazine HCl	Imipramine HCl	0.163	0.520	0.154	1.357	0.214
Loxapine succinate	Chlorpromazine HCl	0.198	0.559	0.277	1.360	0.209
Chlorpromazine HCl	Methiothepin maleate	0.094	0.559	0.413	1.465	0.209

Supplementary Table 4. Small molecule screening data (NT-650)

Category	Parameter	Description
	Type of assay	High throughput drug screen
	Target	Behavioral responses to automated stimuli
	Primary measurement	Raw video whole-plate recordings of fish movement behavior
	Key reagents	DMSO, H2O, library compounds, Singapore strain zebrafish
	Assay protocol	14-minute predefined battery of light and acoustic stimuli
Assay	Additional comments	
	Library size	661 CNS receptor ligands
	Library composition	Neurotransmitter library Containing CNS receptor ligands
	Source	Enzo Life Sciences. Product Number BML-2810
Library	Additional comments	Link to product: https://www.enzo.com/product/screen-well-neurotransmitter-library-10-plate-set/
	Format	96 well plate: 8 DMSO vehicle control, 2-3 H2O vehicle control, 2-3 Eugenol lethal control and 84 screening compounds.
	Concentration(s) tested	10 uM
	Plate controls	DMSO vehicle, H2O vehicle, eugenol lethal control
	Reagent/ compound dispensing system	Beckman Coulter Biomek FXP liquid handler.
	Detection instrument and software	PointGrey Grasshopper GS3-U3-41C6M-C (FLIR Integrated Imaging Solutions).
	Assay validation/QC	Assays were validated on a 16-compound QC set with 10 repeats and plate position randomization
	Correction factors	CD10 to correct for random variation in camera pixel brightness to differentiate between pixel noise and actual zebrafish movement
	Normalization	Simple min/max normalization on a screen-wide level
Screen	Additional comments	Link to camera: https://www.teledynevisionsolutions.com/products/grasshopper3-usb3/?segment=iis&vertical=machine%20vision Link to camera software drivers: https://softwareservices.flir.com/Spinnaker/latest/_spin_view_guide.html Link to code repo: https://github.com/dmyersturnbull/sauronlib Link to instrument facilities: https://pharm.ucsf.edu/smdc/facilities-instrumentation
	Hit criteria	N/A. This assay was primarily used to train models, not for hit finding. See DIVERSet (Table T5) for hit-finding.
	Hit rate	N/A. We trained models on NT-650 and ordered compounds from DIVERSet to look for hits
	Additional assay(s)	N/A
	Confirmation of hit purity and structure	N/A
Post-HTS analysis	Additional comments	

Supplementary Table 5. Small molecule screening data (DIVERSet)

Category	Parameter	Description
	Type of assay	High throughput drug screen
	Target	Behavioral responses to automated stimuli
	Primary measurement	Raw video recordings of fish movement behavior
	Key reagents	DMSO, library compounds, Singapore strain zebrafish
	Assay protocol	14-minute predefined battery of light and acoustic stimuli
Assay	Additional comments	
	Library size	10,000 structurally diverse compounds
	Library composition	Structurally diverse compounds
	Source	ChemBridge DIVERSet 10K compounds
Library	Additional comments	https://chembridge.com/wp-content/uploads/2022/08/ChemBridge-DIVERSet-Libraries.pdf
	Format	96 well plate: 16 DMSO vehicle control and 80 screening compounds
	Concentration(s) tested	10 uM
	Plate controls	DMSO vehicle, eugenol lethal control
	Reagent/ compound dispensing system	Beckman Coulter Biomek FXP liquid handler.
	Detection instrument and software	PointGrey Grasshopper GS3-U3-41C6M-C (FLIR Integrated Imaging Solutions).
	Assay validation/QC	Assays were validated on a 16-compound QC set with 10 repeats and plate position randomization
	Correction factors	CD10 to correct for random variation in camera pixel brightness to differentiate between pixel noise and actual zebrafish movement.
	Normalization	Simple min/max normalization on a screen-wide level
Screen	Additional comments	Link to camera: https://www.teledynevisionsolutions.com/products/grasshopper3-usb3/?segment=iis&vertical=machine%20vision<br>Link to camera software drivers: https://softwareservices.flir.com/Spinnaker/latest/_spin_view_guide.html<br>Link to code repo: https://github.com/dmyersturnbull/sauronlib<br>Link to instrument facilities: https://pharm.ucsf.edu/smdc/facilities-instrumentation
	Hit criteria	Calculated similarity to known-compound readouts
	Hit rate	58% per-query and 22% per-compound hit rate (see Results)
	Additional assay(s)	Radioligand binding assay for in-vitro binding validation (see Methods)
	Confirmation of hit purity and structure	We do not separately verify compound hit purity and structure. The behavioral-based screen readout using AI/ML similarity analysis would also be applicable to blinded hits or mixtures.
Post-HTS analysis	Additional comments	This screen was used for behavioral-similarity-based hit-finding.
