# Supplementary material for: Deep phenotypic profiling of neuroactive drugs in larval zebrafish
Source: Nat Commun. 2024 Nov 17;15:9955. doi: 10.1038/s41467-024-54375-y (PMC11570628; doi:10.1038/s41467-024-54375-y)
Supplement: Supplementary file 2 — Description of Additional Supplementary Files [file 41467_2024_54375_MOESM2_ESM.docx]

**Description of Additional Supplementary Files**

**Supplementary Data 1:** This dataset contains information for the NT-650 and DIVERSet compound libraries. Each file is a CSV with compound information such as identifiers (CIDs) and chemical structures (SMILES). Each CSV also contains additional fields with information specific to the vendor or experimental screen (e.g., plate and well numbers).
