# Supplementary material for: Deep phenotypic profiling of neuroactive drugs in larval zebrafish
Source: Nat Commun. 2024 Nov 17;15:9955. doi: 10.1038/s41467-024-54375-y (PMC11570628; doi:10.1038/s41467-024-54375-y)
Supplement: Supplementary file 4 — Reporting Summary [file 41467_2024_54375_MOESM4_ESM.pdf]

nature portfolio

Corresponding author(s): David Kokel and Michael J. Keiser

Last updated by author(s): Oct 11, 2024

Reporting Summary

Nature Portfolio wishes to improve the reproducibility of the work that we publish. This form provides structure for consistency and transparency in reporting. For further information on Nature Portfolio policies, see our Editorial Policies and the Editorial Policy Checklist.

Statistics

For all statistical analyses, confirm that the following items are present in the figure legend, table legend, main text, or Methods section.

n/a	Confirmed	
[ ]	[x]	The exact sample size (*n*) for each experimental group/condition, given as a discrete number and unit of measurement
[ ]	[x]	A statement on whether measurements were taken from distinct samples or whether the same sample was measured repeatedly
[x]	[ ]	The statistical test(s) used AND whether they are one- or two-sided<br>*Only common tests should be described solely by name; describe more complex techniques in the Methods section.*
[x]	[ ]	A description of all covariates tested
[ ]	[x]	A description of any assumptions or corrections, such as tests of normality and adjustment for multiple comparisons
[ ]	[x]	A full description of the statistical parameters including central tendency (e.g. means) or other basic estimates (e.g. regression coefficient) AND variation (e.g. standard deviation) or associated estimates of uncertainty (e.g. confidence intervals)
[x]	[ ]	For null hypothesis testing, the test statistic (e.g. *F*, *t*, *r*) with confidence intervals, effect sizes, degrees of freedom and *P* value noted<br>*Give P values as exact values whenever suitable.*
[x]	[ ]	For Bayesian analysis, information on the choice of priors and Markov chain Monte Carlo settings
[x]	[ ]	For hierarchical and complex designs, identification of the appropriate level for tests and full reporting of outcomes
[x]	[ ]	Estimates of effect sizes (e.g. Cohen's *d*, Pearson's *r*), indicating how they were calculated

Our web collection on statistics for biologists contains articles on many of the points above.

Software and code

Policy information about availability of computer code

Data collection	Behavioral data were acquired with custom Matlab (Mathworks) and Python scripts. Data was stored in a custom in-house database.
Data analysis	Data analysis was performed using scripts in GraphPad Prism v10, and Python (e.g. numpy (1.16.3), pandas (0.24.2), scipy (1.5.1), sklearn (0.20.3), and seaborn (0.11.1), rdkit (2019.03.2), pytorch (1.1.0)). SEA (Python code) is referenced in Keiser MJ, Roth BL, Armbruster BN, Ernsberger P, Irwin JJ, Shoichet BK. Relating protein pharmacology by ligand chemistry. Nat Biotech 25 (2), 197-206 (2007). Open source code for the study is available at: https://github.com/keiserlab/deepfish

For manuscripts utilizing custom algorithms or software that are central to the research but not yet described in published literature, software must be made available to editors and reviewers. We strongly encourage code deposition in a community repository (e.g. GitHub). See the Nature Portfolio guidelines for submitting code & software for further information.

Data

Policy information about availability of data

All manuscripts must include a data availability statement. This statement should provide the following information, where applicable:

- Accession codes, unique identifiers, or web links for publicly available datasets
- A description of any restrictions on data availability
- For clinical datasets or third party data, please ensure that the statement adheres to our policy

The source data for the Screenwell NT-650 Neurotransmitter and the Chembridge DIVERSet screens are available as a Zenodo repository at https://doi.org/10.5281/zenodo.10652682

Research involving human participants, their data, or biological material

Policy information about studies with human participants or human data. See also policy information about sex, gender (identity/presentation), and sexual orientation and race, ethnicity and racism.

Reporting on sex and gender	N/A
Reporting on race, ethnicity, or other socially relevant groupings	N/A
Population characteristics	N/A
Recruitment	N/A
Ethics oversight	N/A

Note that full information on the approval of the study protocol must also be provided in the manuscript.

Field-specific reporting

Please select the one below that is the best fit for your research. If you are not sure, read the appropriate sections before making your selection.

☒ Life sciences ☐ Behavioural & social sciences ☐ Ecological, evolutionary & environmental sciences

For a reference copy of the document with all sections, see nature.com/documents/nr-reporting-summary-flat.pdf

Life sciences study design

All studies must disclose on these points even when the disclosure is negative.

Sample size	Sample sizes for zebrafish phenotypic screens were chosen based on our ability to generate reproducible and statistically significant differences between positive and negative control groups.
Data exclusions	None.
Replication	All data were collected in replicate. Screens for deep learning model training were performed with n=7-10 replicates per treatment. No replication attempts failed.
Randomization	For all behavioral screens, zebrafish were randomly allocated into 96-well plates from large groups of larvae. For the fully-randomized Screenwell NT-650 screen, a Biomek robot dispensed compounds into wells following randomized layouts created using a custom in-house Python code.
Blinding	Blinding was not used in the screen because compounds were uniformly transferred from the library plates. It is theoretically possible that biases could have been introduced at the pipetting step, for example, if a researcher subconsciously altered pipetting accuracy for certain compounds, but this possibility is unlikely due to the use of calibrated pipettemans. All the behavioral experiments were done with large numbers of randomly assigned animals, in many replicate wells, and on multiple days to provide convincing evidence that reported effects were due to the intended variables, rather than unintended bias. Also, the analyses of large-data sets were uniform, reducing the likelihood of unintentional bias.

Reporting for specific materials, systems and methods

We require information from authors about some types of materials, experimental systems and methods used in many studies. Here, indicate whether each material, system or method listed is relevant to your study. If you are not sure if a list item applies to your research, read the appropriate section before selecting a response.

Materials & experimental systems

n/a	Involved in the study
X	Antibodies
X	Eukaryotic cell lines
X	Palaeontology and archaeology
	X Animals and other organisms
X	Clinical data
X	Dual use research of concern
X	Plants

Methods

n/a	Involved in the study
X	ChIP-seq
X	Flow cytometry
X	MRI-based neuroimaging

Animals and other research organisms

Policy information about studies involving animals; ARRIVE guidelines recommended for reporting animal research, and Sex and Gender in Research

Laboratory animals	Species: Danio rerio. Strain: wild-type zebrafish from Singapore. Age: Zebrafish were studied at 7 days post fertilization, unless otherwise noted in the text.
Wild animals	The study did not involve wild animals.
Reporting on sex	Zebrafish larvae used in these studies have not undergone sexual differentiation at this stage, eliminating sexual dimorphism as a potential confounding factor. Additionally, the larvae are sourced from large group spawnings of wild-type zebrafish, which ensures a genetically diverse and balanced mix of male and female progenitors.
Field-collected samples	The study did not include field-collected samples.
Ethics oversight	Animal husbandry was performed in an AAALAC (Association for Assessment and Accreditation of Laboratory Animal Care International) accredited facility. All zebrafish procedures were approved by UCSF's Institutional Animal Care Use Committee (IACUC), and in accordance with the Guide to Care and Use of Laboratory Animals (National Institutes of Health 1996) and conducted according to established protocols that complied with ethical regulations for animal testing and research.

Note that full information on the approval of the study protocol must also be provided in the manuscript.

Plants

Seed stocks	*Report on the source of all seed stocks or other plant material used. If applicable, state the seed stock centre and catalogue number. If plant specimens were collected from the field, describe the collection location, date and sampling procedures.*
Novel plant genotypes	*Describe the methods by which all novel plant genotypes were produced. This includes those generated by transgenic approaches, gene editing, chemical/radiation-based mutagenesis and hybridization. For transgenic lines, describe the transformation method, the number of independent lines analyzed and the generation upon which experiments were performed. For gene-edited lines, describe the editor used, the endogenous sequence targeted for editing, the targeting guide RNA sequence (if applicable) and how the editor was applied.*
Authentication	*Describe any authentication procedures for each seed stock used or novel genotype generated. Describe any experiments used to assess the effect of a mutation and, where applicable, how potential secondary effects (e.g. second site T-DNA insertions, mosiacism, off-target gene editing) were examined.*
